# Efficient generation of mouse models with the prime editing system

**DOI:** 10.1038/s41421-020-0165-z

**Published:** 2020-04-28

**Authors:** Yao Liu, Xiangyang Li, Siting He, Shuhong Huang, Chao Li, Yulin Chen, Zhen Liu, Xingxu Huang, Xiaolong Wang

**Affiliations:** 1 Key Laboratory of Animal Genetics, Breeding and Reproduction of Shaanxi Province, College of Animal Science and Technology, Northwest A&F University, Yangling, 712100 Shaanxi China; 2 School of Life Science and Technology, ShanghaiTech University, Shanghai, 201210 China; 3 Institute of Neuroscience, CAS Center for Excellence in Brain Science and Intelligence Technology, CAS Key Laboratory of Primate Neurobiology, State Key Laboratory of Neuroscience, Chinese Academy of Sciences, Shanghai, 200031 China; 4 University of Chinese Academy of Sciences, Beijing, 100049 China; 5 CAS Center for Excellence in Molecular Cell Science, Shanghai Institute of Biochemistry and Cell Biology, Chinese Academy of Sciences, Shanghai, 200031 China

**Keywords:** Genomic analysis, Bioinformatics

Dear Editor,

Most genetic diseases in humans are caused by single-nucleotide mutations. Although genome editing with either the CRISPR-based cytosine base editor (CBE)^1^ or the adenine base editor (ABE)^2^ holds great promise for gene correction of C-to-T and A-to-G base substitutions in some genetic diseases^3,4^, both editors are useless for correction of other variants such as base transversion, small insertions and deletions (indels).

The prime editing system, a “search-and replace” genome editing technology, was recently added to the genome editing toolkit^5^. The prime editors (PEs) combine an exogenous CRISPR/Cas9 system and endogenous DNA repair system to achieve an increased range of editing versatility, induces all types of base-to-base conversions out of CBE and ABE (C→T, G→A, A→G, and T→C), small indel, and their combinations. The prime editing system evolved from PE1 to PE3 (PE3b) with stepwise efficiency improvement^5^. The executor of PE1 was constructed by fusing an engineered Cas9 nickase with a reverse transcriptase (M-MLV RTase)^5^, which can target genome sites, nick DNA, and trigger reverse transcription (RT). The executor combining with the engineered prime editing guide RNA (pegRNA) searches for and nicks the target DNA, and thus, new genetic information is encoded into genome by RT. Then, mutations were introduced to M-MLV RTase to improve the editing efficiency of PE1, which is referred to as PE2. Subsequently, in the PE3 system, to further improve editing efficiency, an additional sgRNA is used to induce nick on the non-edited strand to trigger the endogenous mismatch repair pathway^5^.

In comparison with base editors, PE induces base institutions in more extended regions with fewer bystander mutations^5^. With its unique versatility and accuracy, this technology broadens the scope of genome editing and opens a new avenue for targeted mutagenesis and gene correction in many organisms. However, the efficiency of PE was reported only in five different cell types^5^; it has not been investigated in animals. Here, we demonstrate that PE can be employed to generate mutant mice with single-nucleotide substitutions.

We first validated the editing versatility of PEs in human HEK293T cells at eight loci (Supplementary Table S1), including two loci (*RUNX1* and *RNF2*) that were reported by Anzalone et al^5^. PE3 was selected for gene targeting validation, due to its higher editing efficiency compared with PE2^5^. Sanger sequencing revealed that PE3 induced significant base conversions at six (*RUNX1*, *RNF2*, *EMX1*, *VEGFA*, *SRD5A3,* and *KCNA1*) out of eight targeted sites (Supplementary Fig. S1a, b).

PE3 was then used to induce point mutations in the X-linked androgen receptor (*Ar*) gene and the homeobox protein Hox-D13 (*Hoxd13*) gene in mouse neuro-2a (N2a) cells^6,7^. Both targeted mutations in mice are homologous to human variants associated with clinical diseases in ClinVar^8,9^. pegRNAs and nick-editing sgRNAs targeting these two genes were designed (Supplementary Table S2). We designed pegRNAs starting with a primer binding site (PBS) length of 13 nt and an RT template length of ~13–15 nt. Nicks were positioned 3′ of the edit ~40–60 bp from the pegRNA-induced nick. Sanger sequencing revealed that PE3 efficiently (~8–40%) mediated base transversions at three target sites of *Hoxd13* and *Ar* (pegHoxd13-1 for G to C, pegHoxd13-2 for G to T, pegAr-2 for G to T) (Fig. 1a; Supplementary Fig. S2a–d). Next, to optimize the performance of pegRNAs, we systematically evaluated pegRNAs with RT template lengths of 10–20 nt and a PBS length of 13 nt for pegHoxd13-1, pegHoxd13-2, and pegAr-2 in N2a cells. Targeted deep sequencing revealed that the editing efficiency of pegRNAs with RT template lengths ranging from 14 to 17 nt were stably efficient in *Hoxd13* and *Ar*, respectively (Fig. 1c; Supplementary Fig. S3a). Then, using the efficient RT template lengths of 14–17 nt, we systematically evaluated pegRNAs in the context of PBS lengths between 10 and 17 nt. Targeted deep sequencing revealed that pegRNAs with a PBS length of 16, 13, and 12 nt for pegHoxd13-1, pegHoxd13-2, and pegAr-2, respectively, had the highest efficiencies; these pegRNAs were used for our in vivo study (Fig. 1b, d; Supplementary Fig. S3b). Anzalone et al. reported that the PE3b system can be applied to the edit lying within a second protospacer to induce fewer indels^5^. Accordingly, we designed nick sgRNA for *Hoxd13* based on these parameters and found that the editing efficiency of PE3b was significantly lower than that mediated by PE3 in *Hoxd13* (Supplementary Fig. S4), we then selected PE3 for the further analysis.

**Fig. 1:**
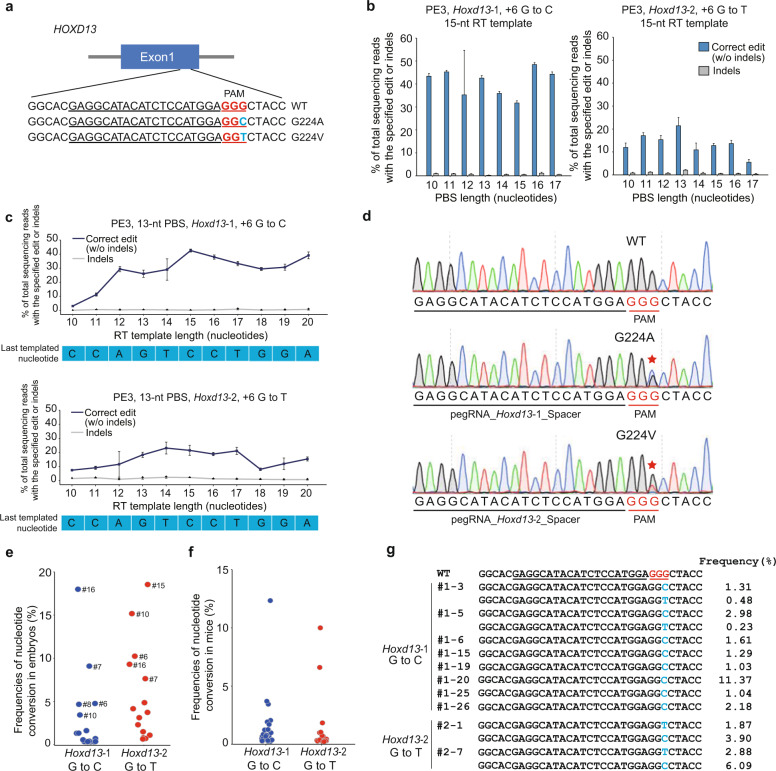
PE3-mediated efficient base transversion in vitro and in vivo. **a** Schematic diagram of target site at the *HOXD13* locus. The PAM sequences and the spacer sequences of pegRNA are underlined in red and black, respectively; the nucleotide substitutions are marked in blue. The corresponding targeted codons are shown on the right of sequence. **b** Editing efficiency and indel generation by PE3 at the +6 position of *HOXD13* using pegRNAs containing 15-nt RT templates and PBS sequences ranging from 10 to 17 nt in N2a cells. Indels (gray column) are plotted for comparison. **c** PE3-mediated base transversion editing efficiency and indels at the +6 position of *HOXD13* in mouse N2a cells as a function of RT template length. Indels (gray line) are plotted for comparison. The sequence below the graph shows the last nucleotide template for synthesis by the pegRNA. **d** Sanger sequencing chromatograms at the *HOXD13* site in N2a cells. The PAM sequence and spacer sequence of pegRNA are underlined in red and black, respectively. Asterisks indicate the desired editing. **e** Frequencies of nucleotide conversions in mouse embryos. PCR amplicons from the target regions in *Hoxd13* were analyzed by targeted deep sequencing. Each dot indicates one individual embryo; embryo id was marked for the embryos with top five nucleotide conversion frequency. **f** Frequencies of nucleotide conversions in mice. PCR amplicons from the target regions in *Hoxd13* were analyzed by targeted deep sequencing. Each dot indicates one individual mouse. **g** Genotypes of the two *Hoxd13* sites in mice by targeted deep sequencing. Alignment of sequences derived from deep sequencing in ten mutant mice. The target site is highlighted in blue. pegRNA spacer sequence are underlined in black. PAM sequences are underlined and marked in red.

To edit mouse embryos with PE3, pCMV-PE2-encoding mRNA and corresponding nick sgRNA were co-injected with different pegRNAs into one-cell embryos. For *Hoxd13*, nucleotide conversions were observed in 8 out of 18 (44%) and 12 out of 16 (75%) blastocysts, respectively, with mutation frequencies ranging from 1.1 to 18.5% (Fig. 1e). We observed a low frequency of indels in injected embryos (Supplementary Fig. S5a). Interestingly, we observed not only desirable transversion at the target sites, but also a mixture of nucleotide conversions at these sites (Supplementary Fig. S6), implying a relative low fidelity of the PE3 system in murine embryos that the PE is in need of improved fidelity. Moreover, we observed much lower editing efficiency in embryos for the *Ar* gene (Supplementary Fig. S7), suggesting that screening of pegRNAs in vitro is essential before conducting in vivo studies.

We next delivered mouse embryos that were injected with pCMV-PE2-encoding mRNA and pegRNAs for *Hoxd13* into surrogate mothers. Targeted deep sequencing indicated that eight mutant mice out of 30 (*Hoxd13-*1, editing efficiency of G to C > 1%) and two mutant mice out of 19 (*Hoxd13*-2, editing efficiency of G to T > 1%) carried mutations at the targeted loci (Fig. 1f, g). Furthermore, the editing efficiency in mutant mice is equivalent to that in prime-edited plants^10^. Together with indel frequency data in embryos and mutant mice (Supplementary Fig. S5), we revealed a low level of indels in the PE system in vivo. As expected, targeted deep sequencing revealed that the editing efficiency varied in the examined nine tissues (heart, liver, spleen, lung, kidney, brain, testis, intestine, and toe) of the two mutant mice (#1–5 and #2–14) (Supplementary Fig. S8), indicating that the somatic mosaicism and allelic complexity were induced by zygotic injection. As observed in mouse embryos, we found existence of varied frequencies of undesirable base changes at the two target sites (Fig. 1g).

To determine off-target mutations in PE-edited mice, we identified, using Cas-OFFinder^11^, ten and six putative off-target sites with up to three-nucleotide mismatches and NGG PAM sites for pegRNA and nick sgRNA, respectively. Targeted deep sequencing with genomic DNA from five PE-edited mice (#1–5, #1–6, #1–20, #2–14, and #2–18) revealed undetectable off-target mutations at all 16 sites (Supplementary Fig. S9a, b).

To comprehensively investigate off-target sites throughout the genome, we conducted whole-genome sequencing for the *Hoxd13* mutant mice #1–26 (G-to-C) and #2–1 (G-to-T). A total of 3,745,168 and 3,840,538 SNPs were identified for #1–26 and #2–1, respectively (Supplementary Fig. S10). After filtering out naturally-occurring variants in the dbSNPs database and in two wild-type mice, we picked out the variants with G-to-H (H indicates A/C/T) and C-to-H substitutions, and examined whether the remaining SNPs located at the putative off-target sites. Of 3805 predicted off-target sites, no base substitutions were uniquely found in #1–26 and #2–1 (Supplementary Fig. S10). These results demonstrate that the PE3-mediated base conversion is highly specific in vivo.

In sum, we present here the first report of using PEs to generate targeted base conversion mutations in animals. We validate the prime editing system in human cells and demonstrate the versatility of PEs in mice in vivo, though there is a higher frequency of unwanted mutations at target loci. These data support the clinical potential of PEs in correcting a broad range of mutations for genetic diseases.

## Supplementary information

Supplementary information

## Supplementary information

**Supplementary Information** accompanies the paper at (10.1038/s41421-020-0165-z).
