# Supplementary material for: Efficient generation of mouse models with the prime editing system
Source: Cell Discov. 2020 Apr 28;6:27. doi: 10.1038/s41421-020-0165-z (PMC7186222; doi:10.1038/s41421-020-0165-z)
Supplement: Supplementary file 1 — Supplementary information [file 41421_2020_165_MOESM1_ESM.pdf]

SUPPLEMENTARY INFORMATION

Material and Methods

Animals

Mice were maintained in an Assessment and Accreditation of Laboratory Animal Care that were credited specific pathogen free facility under a 12 h dark-light cycle. B6D2F1 (C57BL/6 × DBA/2) and ICR mouse strains were used as embryo donors and foster mothers, respectively. All the animal experiments were approved by the Animal Care and Use Committee of the Institute of Neuroscience, Chinese Academy of Sciences, Shanghai, China.

Plasmid construction

pCMV-PE2 was purchased from Addgene (Addgene, 132775). The pegRNA plasmid was constructed according to the reported protocol partially[1]. pegRNA plasmid backbone was amplified from pGL3-U6-sgRNA-EGFP (Addgene, 107721) using Phanta® Max Super Fidelity DNA Polymerase (Vazyme) (Supplementary Table S3). Then pegRNA plasmid backbone amplicon was cut by BsaI-HFv2 (NEB) for overhangs. Spacer oligos (the top strand oligo includes the 5' ACCG and 3' GTTTT overhangs, and the bottom strand oligo includes the 5' CTCTAAAAC overhang), pegRNA 3' extension oligos (the top strand oligo includes the 5' GTGC overhang and the bottom strand oligo includes 5' AAAA overhang), sgRNA scaffold oligo (featuring compatible overhangs) were synthesized (Supplementary Table S3). Annealing top and bottom oligos. Phosphorylating sgRNA scaffold sequence with T4 PNK (NEB), spacer sequence, 3' extension sequence and sgRNA scaffold sequence were cloned into backbone of pegRNA expression vector with T4 DNA ligase (NEB). For construction of sgRNAs, oligos were synthesized, annealed, and cloned into BsaI site of the pGL3-U6-sgRNA-mCherry expression vector.

Cell culture and transfection

HEK293T and Neuro-2a (N2a) cells were cultured in Dulbecco's Modified Eagle Medium (Gibco), supplemented with 10% fetal bovine serum (FBS) (v/v) (Gemini) and incubated at 37 °C with 5% CO_2. For plasmid transfection, cells were seeded on poly-D-lysine-coated 24-well plates and transfected at approximately 70% confluence using EZ Trans (Shanghai Life iLab) according to the manufacturer's protocols. A total of 900 ng PE2, 300 ng pegRNAs and 100 ng corresponding nick sgRNA were transfected into cells per well. All pegRNAs and sgRNAs used in this study are listed in Supplementary Table S1 & S2. 72 h after transfection, GFP and mCherry double-positive cells were collected from Fluorescence Activating Cell Sorter (FACS).

Genomic DNA extraction and genotyping

Genomic DNA of GFP and mCherry double-positive cells were extracted using QuickExtract™ DNA Extraction Solution (Lucigen) according to manufacturer's protocols. Genomic DNA of mouse was extracted by the phenol-chloroform method. The isolated DNA was PCR-amplified with Phanta® Max Super-Fidelity DNA Polymerase (Vazyme). Primers used were listed in Supplementary Table S4.

Estimation of editing frequency

The online software EditR (https://moriaritylab.shinyapps.io/editr_v10/)[2] was applied to estimate editing frequency using Sanger sequencing.

***In vitro* transcription**

The PE2 plasmid was linearized by PmeI enzyme (NEB) and *in vitro* transcription was performed using the T7 Ultra Kit (Ambion). RNA was purified by the Mini Kit (Qiagen) according to the manufacturer's protocols. All *in vitro* transcription templates of pegRNAs and sgRNAs used for *in*

vitro transcription were amplified from their PGL3-U6 expression vector using corresponding primers. Then, the pegRNAs and sgRNAs were transcribed *in vitro* by the MEGA shortscript Kit (Ambion) and purified by the MEGA clear Kit (Ambion) according to the manufacturer's protocols. Primers used for *in vitro* transcription were listed in Supplementary Table S5.

Microinjection of mouse zygotes

C57BL/6J × DBA/2J F1 (B6D2F1) female mice (4 weeks of age) were superovulated and mated with B6D2F1 male mice. Mouse zygotes were collected from the oviducts. The mRNA mixture containing pegRNA (50 ng/μl), nick-sgRNA (16.7 ng/μl) and PE2 mRNA (100 ng/μl) was injected into the cytoplasm of zygotes in a droplet of M2 medium containing 5 μg/ml cytochalasin B (CB) with a piezo-driven micromanipulator (Primetech). The injected zygotes were cultured in KSOM medium at 37°C under 5% of CO_2 and transferred to oviducts of pseudopregnant females at 0.5 days post copulation.

Targeted deep sequencing

The potential off-target sites of *Hoxd13*-1 and *Hoxd13*-2 were predicted by Cas-OFFinder[3] (http://www.rgenome.net/cas-offinder). The on-target and off-target sites were amplified from genomic DNA using Phanta® Max SuperFidelity DNA Polymerase (Vazyme). Primers used for deep sequencing were listed in Supplementary Table S4. The paired-end sequencing of PCR amplicons was performed by Illumina Nextseq 500 (2 × 150) platform at CAS-MPG Partner Institute for Computational Biology Omics Core, Shanghai, China.

Whole genome sequencing

Two mutant mice #1-26 and #2-1 were subject to whole genome sequencing (WGS). To construct the WGS library, 1 μg of genomic DNA extracted from tails was fragmented to around 300 bp by ultrasonication using a Covaris S2 system. Then, the sheared DNA fragments were used for library

construction and the library were sequenced using Hiseq X Ten platform (Illumina) as paired-end 150 base reads at the CAS-MPG Partner Institute for Computational Biology Omics Core, Shanghai, China. The WGS data of two wild-type mice have the same genetic background with the founders was used in this study: wild-type_1 (sequenced in the present study) and wild-type_2 (sequenced previously[4]).

The raw sequencing reads were first filtered to remove low quality paired reads with the following criteria: (1) a sequencing quality of <3, and (2) reads with residual length of <40 bases after the adaptor sequences were trimmed. All reads that passed the quality control procedures were converted into FASTQ files. All cleaned reads were mapped to the mouse reference genome (GRCm38/mm10) using BWA v0.7.13[5] with default parameters. Variants were identified by Genome Analysis Toolkit (GATK v3.7)[6] HaplotypeCaller and the following criteria were applied to all SNPs: (1) Sequencing depth (for each individual) > 1/4 × and <4 ×; (2) RMS mapping quality (MQ) > 40.0; (3) Phred-scaled P value using Fisher's exact test to detect strand bias < 60; (4) Z-score from the Wilcoxon rank sum test of Alt vs. Ref read MQs (MQRankSum) > −12.5; and (5) Z-score from the Wilcoxon rank sum test of Alt vs. Ref read position bias (ReadPosRankSum) > −8. After filtering out variants in the dbSNP database and also the variants in the two wild-types, we picked out the variants with C and G converted to other bases among the remaining variants. We compared these remaining variants with the putative off-target sites, which were predicted by Cas-OFFinder[3] considering mismatch up to 5 mismatches with NGG PAM.

Statistical analysis

Targeted deep-sequencing data were processed with the CRISPResso2[7] algorithm in batch mode using default parameters. Data are expressed as mean values ± s.e.m. Statistical analyses were performed using paired-samples Student's t tests in R environment[8] when applicable. Differences were considered significant at $p < 0.01$ or $p < 0.05$ as indicated.

Data availability

WGS and targeted amplicon sequencing data has been deposited at the SRA repository under

Bioproject number PRJNA604409. Plasmids used in the study are available through Addgene, and annotated DNA sequences for all constructs are available upon request.

References:

1 Anzalone, A. V. *et al.* Search-and-replace genome editing without double-strand breaks or donor DNA. *Nature* **576**, 149-157, doi:10.1038/s41586-019-1711-4 (2019).

2 Kluesner, M. G. *et al.* EditR: A Method to Quantify Base Editing from Sanger Sequencing. *CRISPR J* **1**, 239-250, doi:10.1089/crispr.2018.0014 (2018).

3 Bae, S., Park, J. & Kim, J. S. Cas-OFFinder: a fast and versatile algorithm that searches for potential off-target sites of Cas9 RNA-guided endonucleases. *Bioinformatics* **30**, 1473-1475, doi:10.1093/bioinformatics/btu048 (2014).

4 Li, J. *et al.* Efficient base editing in G/C-rich regions to model androgen insensitivity syndrome. *Cell Res* **29**, 174-176, doi:10.1038/s41422-018-0133-4 (2019).

5 Li, H. & Durbin, R. Fast and accurate short read alignment with Burrows-Wheeler transform. *Bioinformatics* **25**, 1754-1760, doi:10.1093/bioinformatics/btp324 (2009).

6 McKenna, A. *et al.* The Genome Analysis Toolkit: a MapReduce framework for analyzing next-generation DNA sequencing data. *Genome Res* **20**, 1297-1303, doi:10.1101/gr.107524.110 (2010).

7 Clement, K. *et al.* CRISPResso2 provides accurate and rapid genome editing sequence analysis. *Nat Biotechnol* **37**, 224-226, doi:10.1038/s41587-019-0032-3 (2019).

8 Team, R. C. R: a language and environment for statistical computing. https://www.r-project.org (2018).

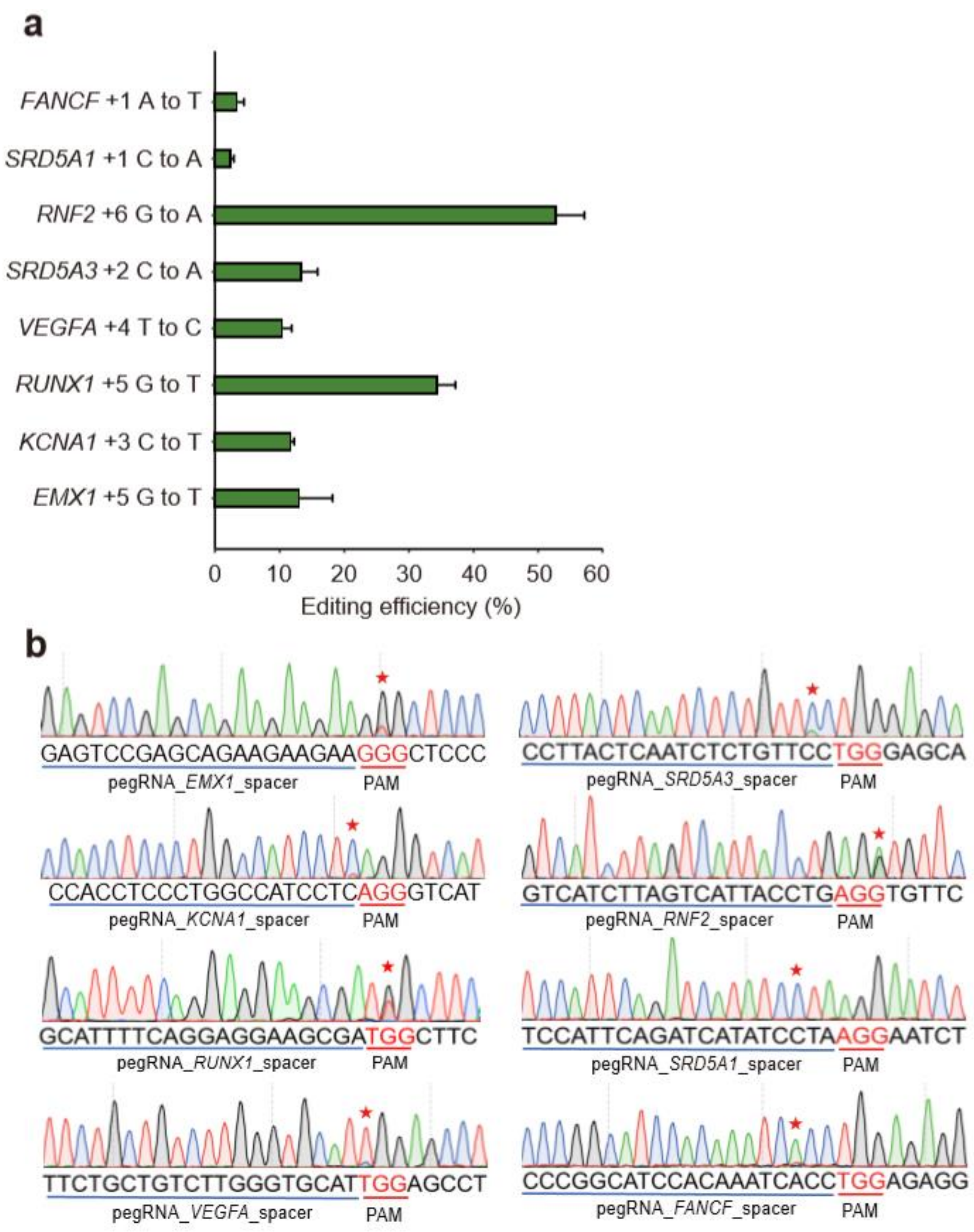

Supplementary Fig. S1 Prime editing in human HEK293T cells.

(a) Editing efficiency of PE3 at eight sites (*RNF2*, *RUNX1*, *SRD5A1*, *SRD5A3*, *EMX1*, *FANCF*, *VEGFA*, and *KCNA1*). The editing efficiency was calculated using EditR. Data from three independent replicates are shown as means ± s.e.m.

(b) Sanger sequencing chromatograms of PE3-mediated editing in human HEK293T cells. The PAM sequence and spacer sequence of pegRNA are underlined in red and blue, respectively. Red stars indicate the targeted bases.

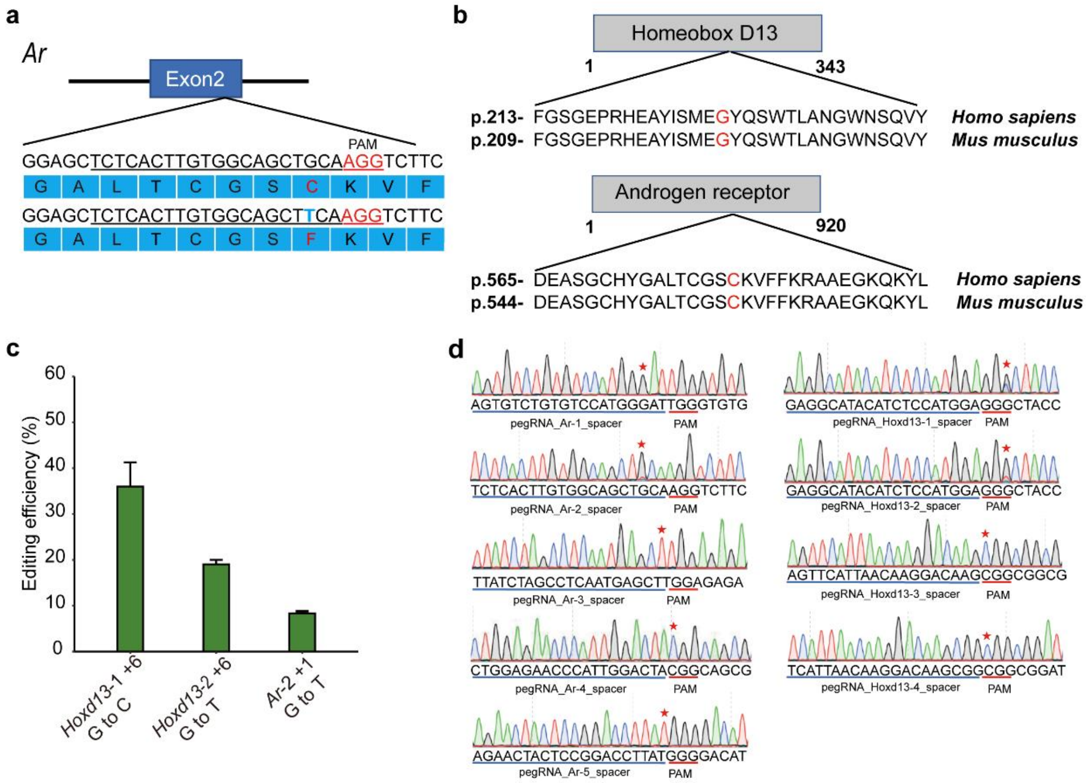

Supplementary Fig. S2 The targeted sequences at the *Ar* and *Hoxd13* loci.

(**a**) Schematic diagram of target site at the *Ar* locus. The PAM sequences and the spacer sequences of pegRNA are underlined in red and black, respectively; the nucleotide substitutions are marked in blue. The corresponding targeted codons in red.

(b) Schematic illustration shows the localization of mutations associated to clinical diseases on Androgen receptor protein and Homeobox D13 protein of human. Lower parts of each illustration show the alignments of the conserved amino acid sequence from *H. sapiens* to *M. musculus*. The targeted amino acid sites in this study were highlighted in red.

(c) Editing efficiency of PE3 at three sites (*Hoxd13*-1, *Hoxd13*-2, and *Ar*-2)**.** The editing efficiency was calculated using EditR. Data from three independent replicates are shown as means ± s.e.m.

(d) Sanger sequencing chromatograms of PE3-mediated editing in N2a cells. The PAM sequence and spacer sequence of pegRNA are underlined in red and blue, respectively. Asterisks indicate the targeted bases.

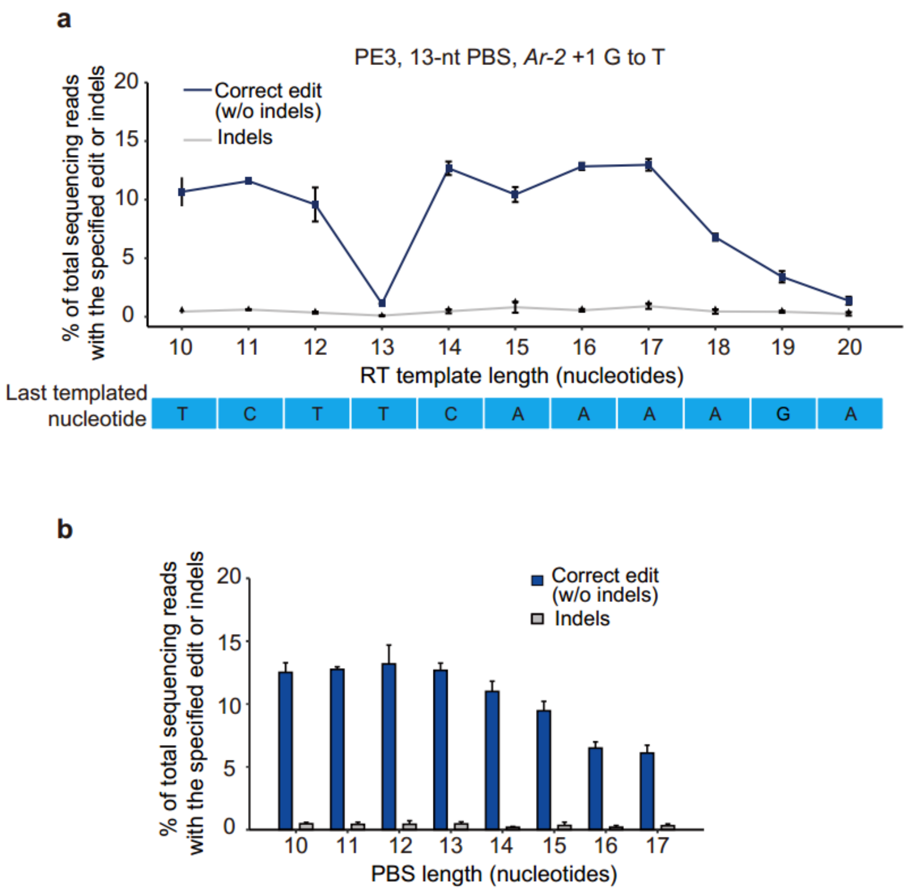

Supplementary Fig. S3 Editing efficiency of PE3 at the *Ar* locus using pegRNAs with different length of PBS and RT.

(**a**) PE3-mediated base transversion editing efficiency and indels at the +1 position of *Ar* in N2a cells as a function of RT template length. Indels (gray line) are plotted for comparison. The sequences below the graph indicate the last nucleotide templated for synthesis by the pegRNA. (**b**) Editing efficiency and indels generation by PE3 at the +1 position of *Ar* using pegRNAs containing 14-nt RT templates and a PBS sequences ranging from 10 - 17 nt in N2a cells. Indels (gray line) are plotted for comparison.

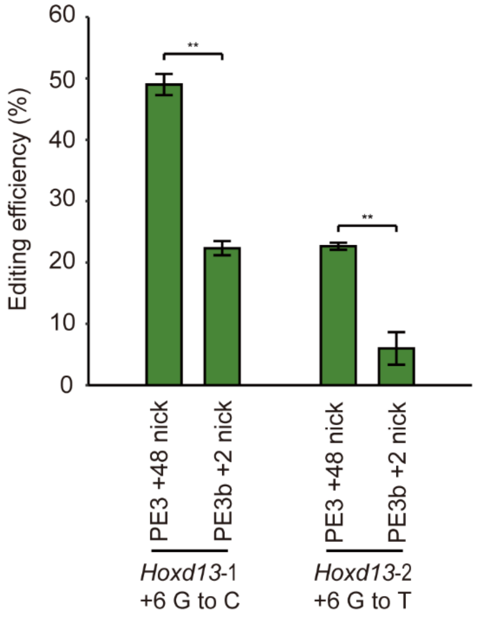

Supplementary Fig. S4 Editing efficiency of PE3b at the *Hoxd13* loci.

Comparison of editing efficiencies with PE3 and PE3b at the *Hoxd13* loci. The editing efficiency was calculated using EditR. Data from three independent replicates are shown as means ± s.e.m. **, $P < 0.01$.

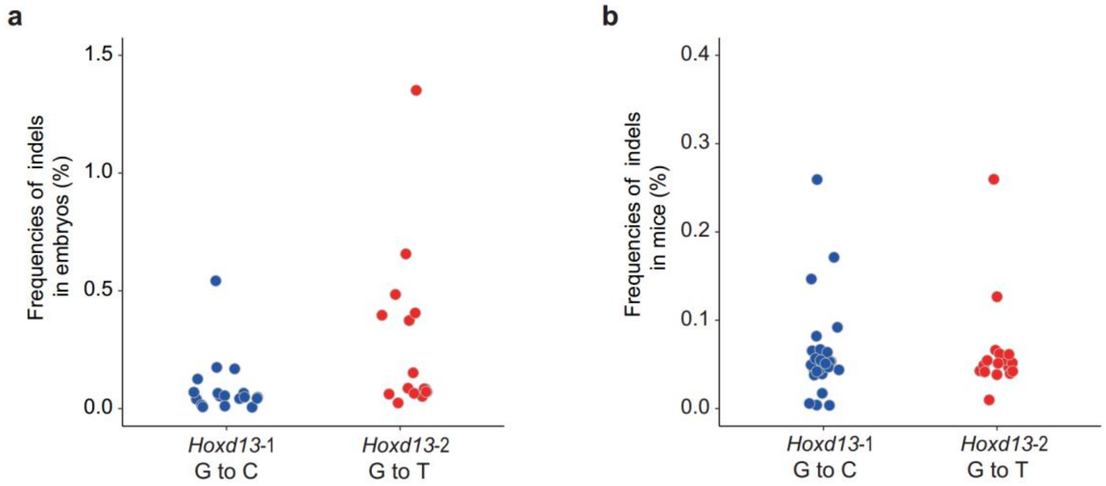

Supplementary Fig. S5 Indel frequency in embryos and mice.

(a) Indel frequency in injected embryos. PCR amplicons from the target regions in *Hoxd13* were analyzed by targeted deep sequencing. Each dot indicates one individual embryo.

(b) Indel frequency in mice. PCR amplicons from the target regions in *Hoxd13* were analyzed by targeted deep sequencing. Each dot indicates one individual.

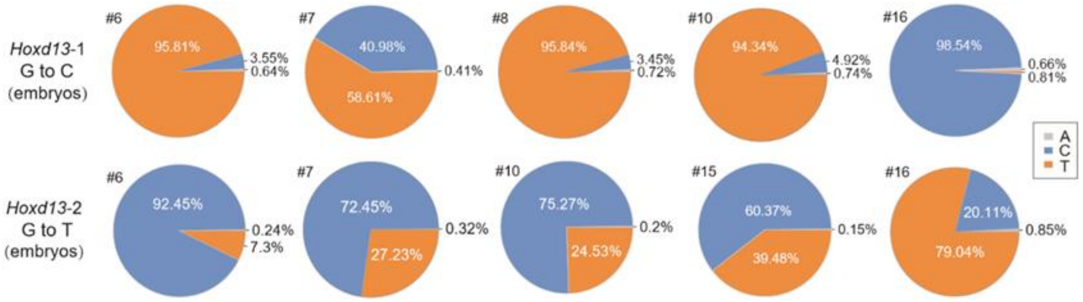

Supplementary Fig. S6 Distribution of mutations introduced by PE3 in mice embryos with the top 5 editing efficiency.

The frequencies of nucleotide conversions in these murine embryos are labeled in Fig. 1e, respectively.

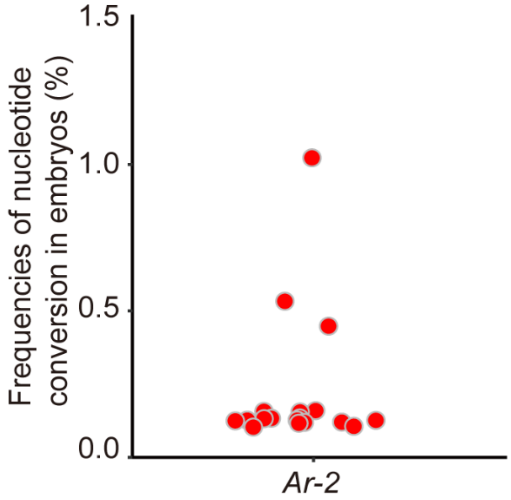

Supplementary Fig. S7 Editing efficiency of PE3 at the *Ar* loci in mouse embryos.

Frequencies of nucleotide conversions in murine embryos. PCR amplicons from the target regions in the *Ar-2* site were analyzed by targeted deep sequencing. Each dot indicates one individual embryo.

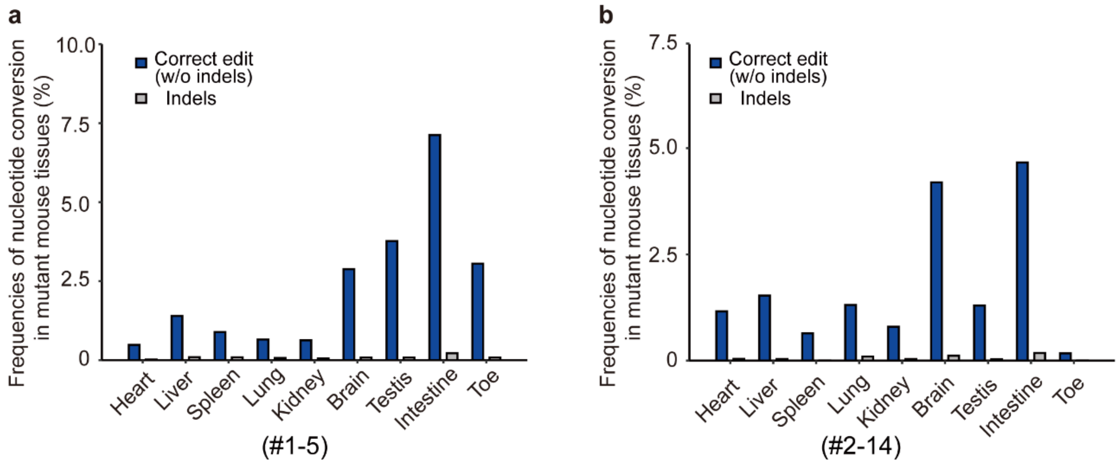

Supplementary Fig. S8 Editing efficiency in the tissues of two mutant mice.

The individual PE3-mediated editing efficiency at the *Hoxd13* site in different tissues of founder mouse #1-5 (**a**) and #2-14 (**b**) at day 14. Data are analyzed by targeted deep sequencing.

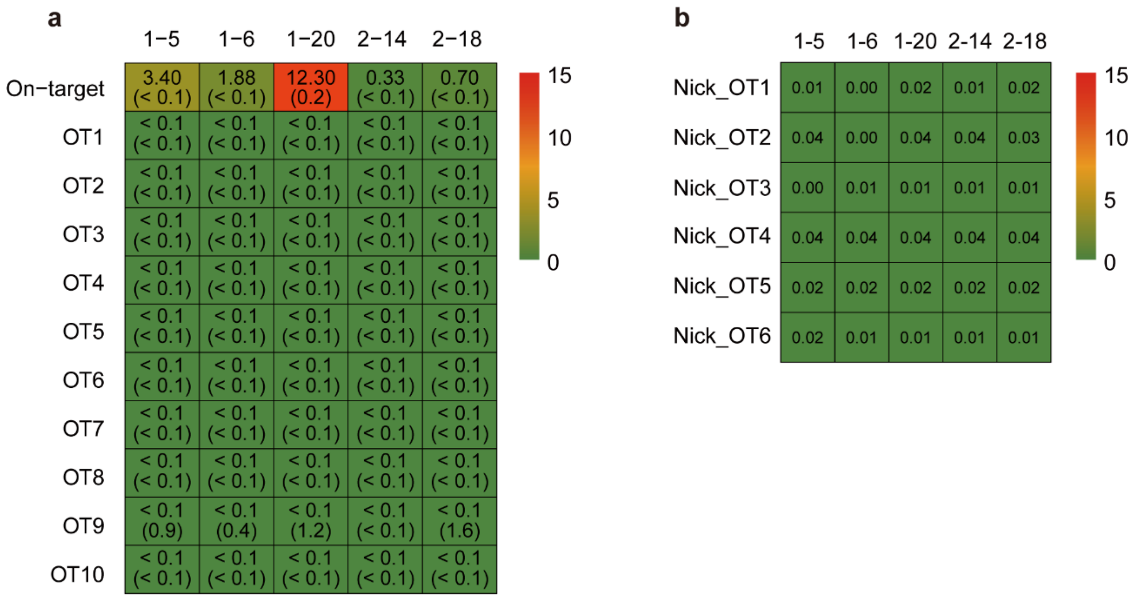

Supplementary Fig. S9 Analysis of off-target in mutant mice by deep sequencing.

(**a**) The on-target and off-target editing efficiencies and indel efficiencies (in parenthesis) in five PE3 mutant mice. The potential off-target sites were predicted by Cas-OFFinder, and were assessed by targeted deep-sequencing.

(**b**) The indel efficiencies of nick sgRNA in five PE3 mutant mice. The potential off-target sites were predicted by Cas-OFFinder, and were assessed by targeted deep-sequencing.

	#1-26	#2-1
**Sequencing coverage**	26.64×	31.05×
**Total SNPs**	3,745,168	3,840,538
**Excluding SNPs in dbSNPs**	201,229	241,408
**Excluding SNPs in wildtypes**	37,005	31,267
**C>A SNPs**	2587	2097
**C>T SNPs**	5847	5097
**C>G SNPs**	1546	1454
**G>A SNPs**	5789	4980
**G>T SNPs**	2480	2025
**G>C SNPs**	1614	1384
**Off-target sequences (3805 sites, up to 5-bp mismatch with NGG PAM)**	0	0

Supplementary Fig. S10 Summary of whole genome sequencing analysis.

The two mutant mice were sequenced with high coverage and 3,745,168 and 3,840,538 SNPs were identified in #1-26 and #2-1, respectively. After filtering out SNPs in the dbSNPs database, and also variants in two wildtype mice (the same genetic background with the mutant mice), 37,005 and 31,267 SNPs were remained. The number of C/G convert to H was showed. By comparison these variants to the 3805 predictive off-target sites, no off-target editing was identified in the two mutant mice.

Supplementary Table S1 Sequences of pegRNAs and sgRNAs used in human HEK293T cells.

**pegRNA**	**spacer sequence**	**3'extension sequence**	**PBS length (nt)**	**RT template length (nt)**
pegRNF2_+6GtoA	GTCATCTTAGTCATTACCTG	AACGAACATCTCAGGTAATGACTAAGATG	15	14
pegRUNX1__+5GtoT	GCATTTTCAGGAGGAAGCGA	TGTCTGAAGCAATCGCTTCCTCCTGAAAAT	15	15
pegEMX1_+5GtoT	GAGTCCGAGCAGAAGAAGAA	ATGGGAGCACTTCTTCTTCTGCTCGGAC	15	13
pegFANCF_+1AtoT	CCCGGCATCCACAAATCACC	GCTCCCCTCTCCAGGAGATTTGTGGATGCCG	15	16
pegKCNA1_+3CtoT	CCACCTCCCTGGCCATCCTC	AAGCGGATGACCCTAAGGATGGCCAGGGAG	13	17
pegVEGFA_+4TtoC	TTCTGCTGTCTTGGGTGCAT	AAGGCAAGGCTCCGATGCACCCAAGACAGCAG	15	17
pegSRD5A1_+1CtoA	TCCATTCAGATCATATCCTA	TTTCTGAGATTCCTTATGATATGATCTGAATG	15	17
pegSRD5A3_+2CtoA	CCTTACTCAATCTCTGTTCC	GAAAAGGTGCTCCCAGTAACAGAGATTGAGTAA	15	17
**nicking sgRNA**	**spacer sequence**			
RNF2_+41	GTCAACCATTAAGCAAAACAT			
RUNX1_+38	GATGAAGCACTGTGGGTACGA			
EMX1_+53	ACATCGATGTCCTCCCCAT			
FANCF_-3	TCCCCTCTCCAGGAGATTTG			
KCNA1_+1	GCAAGCGGATGACCCTAAGGA			
VEGFA_43	CAGGGCACGACCGCTTACCT			
SRD5A1_+48	CACTTTCTGTACGTACCCCT			
SRD5A3_+70	AGCCCAGGGAGTCCTTACCC			

Supplementary Table S2 Sequences of pegRNAs and sgRNAs used in mouse N2a cells.

**pegRNA**	**spacer sequence**	**3'extension sequence**	**PBS length (nt)**	**RT template length (nt)**
pegHoxd13-1_+6GtoC	GAGGCATACATCTCCATGGA	GACTGGTAGCGCTCCATGGAGATGTATG	13	15
pegHoxd13-2_+6GtoT	GAGGCATACATCTCCATGGA	GACTGGTAGCACTCCATGGAGATGTATG	13	15
pegHoxd13-3_+4CtoG	AGTTCATTAACAAGGACAAG	GATCCGCCGCCCCTTGTCCTTGTTAATG	13	15
pegHoxd13-4_+1CtoG	TCATTAACAAGGACAAGCGG	TGAGATCCGCCGCCCCTTGTCCTTGTTA	13	15
pegAr-1_+1GtoT	AGTGTCTGTGTCCATGGGAT	TCCACACCCAATACCATGGACACAGA	13	13
pegAr-2_+1GtoT	TCTCACTTGTGGCAGCTGCA	AAGAAGACCTTGAAGCTGCCACAAGT	13	13
pegAr-3_+3TtoG	TTATCTAGCCTCAATGAGCT	GCCTCTCTCCACGCTCATTGAGGCTAG	13	14
pegAr-4_+4CtoA	CTGGAGAACCCATTGGACTA	GGCGCTGCCTTAGTCCAATGGGTTCT	13	13
pegAr-5_+3TtoA	AGAACTACTCCGGACCTTAT	GCATGTCCCCTTAAGGTCCGGAGTAG	13	13
pegHoxd13-1-RT10	GAGGCATACATCTCCATGGA	GTAGGCCTCCATGGAGATGTATG	13	10
pegHoxd13-1-RT11	GAGGCATACATCTCCATGGA	GGTAGGCCTCCATGGAGATGTATG	13	11
pegHoxd13-1-RT12	GAGGCATACATCTCCATGGA	TGGTAGGCCTCCATGGAGATGTATG	13	12
pegHoxd13-1-RT13	GAGGCATACATCTCCATGGA	CTGGTAGGCCTCCATGGAGATGTATG	13	13
pegHoxd13-1-RT14	GAGGCATACATCTCCATGGA	ACTGGTAGGCCTCCATGGAGATGTATG	13	14
pegHoxd13-1-RT15	GAGGCATACATCTCCATGGA	GACTGGTAGGCCTCCATGGAGATGTATG	13	15
pegHoxd13-1-RT16	GAGGCATACATCTCCATGGA	GGACTGGTAGGCCTCCATGGAGATGTATG	13	16
pegHoxd13-1-RT17	GAGGCATACATCTCCATGGA	AGGACTGGTAGGCCTCCATGGAGATGTATG	13	17
pegHoxd13-1-RT18	GAGGCATACATCTCCATGGA	CAGGACTGGTAGGCCTCCATGGAGATGTATG	13	18
pegHoxd13-1-RT19	GAGGCATACATCTCCATGGA	CCAGGACTGGTAGGCCTCCATGGAGATGTATG	13	19
pegHoxd13-1-RT20	GAGGCATACATCTCCATGGA	TCCAGGACTGGTAGGCCTCCATGGAGATGTATG	13	20
pegHoxd13-1-PBS10	GAGGCATACATCTCCATGGA	GACTGGTAGGCCTCCATGGAGATGT	10	15
pegHoxd13-1-PBS11	GAGGCATACATCTCCATGGA	GACTGGTAGGCCTCCATGGAGATGTA	11	15
pegHoxd13-1-PBS12	GAGGCATACATCTCCATGGA	GACTGGTAGGCCTCCATGGAGATGTAT	12	15
pegHoxd13-1-PBS13	GAGGCATACATCTCCATGGA	GACTGGTAGGCCTCCATGGAGATGTATG	13	15
pegHoxd13-1-PBS14	GAGGCATACATCTCCATGGA	GACTGGTAGGCCTCCATGGAGATGTATGC	14	15
pegHoxd13-1-PBS15	GAGGCATACATCTCCATGGA	GACTGGTAGGCCTCCATGGAGATGTATGCC	15	15
pegHoxd13-1-PBS16	GAGGCATACATCTCCATGGA	GACTGGTAGGCCTCCATGGAGATGTATGCCT	16	15
pegHoxd13-1-PBS17	GAGGCATACATCTCCATGGA	GACTGGTAGGCCTCCATGGAGATGTATGCCTC	17	15
pegHoxd13-2-RT10	GAGGCATACATCTCCATGGA	GTAGACCTCCATGGAGATGTATG	13	10
pegHoxd13-2-RT11	GAGGCATACATCTCCATGGA	GGTAGACCTCCATGGAGATGTATG	13	11
pegHoxd13-2-RT12	GAGGCATACATCTCCATGGA	TGGTAGACCTCCATGGAGATGTATG	13	12
pegHoxd13-2-RT13	GAGGCATACATCTCCATGGA	CTGGTAGACCTCCATGGAGATGTATG	13	13
pegHoxd13-2-RT14	GAGGCATACATCTCCATGGA	ACTGGTAGACCTCCATGGAGATGTATG	13	14
pegHoxd13-2-RT15	GAGGCATACATCTCCATGGA	GACTGGTAGACCTCCATGGAGATGTATG	13	15
pegHoxd13-2-RT16	GAGGCATACATCTCCATGGA	GGACTGGTAGACCTCCATGGAGATGTATG	13	16
pegHoxd13-2-RT17	GAGGCATACATCTCCATGGA	AGGACTGGTAGACCTCCATGGAGATGTATG	13	17
pegHoxd13-2-RT18	GAGGCATACATCTCCATGGA	CAGGACTGGTAGACCTCCATGGAGATGTATG	13	18
pegHoxd13-2-RT19	GAGGCATACATCTCCATGGA	CCAGGACTGGTAGACCTCCATGGAGATGTATG	13	19
pegHoxd13-2-RT20	GAGGCATACATCTCCATGGA	TCCAGGACTGGTAGACCTCCATGGAGATGTATG	13	20
pegHoxd13-2-PBS10	GAGGCATACATCTCCATGGA	GACTGGTAGACCTCCATGGAGATGT	10	15

pegHoxd13-2-PBS11	GAGGCATACATCTCCATGGA	GACTGGTAGACCTCCATGGAGATGTA	11	15
pegHoxd13-2-PBS12	GAGGCATACATCTCCATGGA	GACTGGTAGACCTCCATGGAGATGTAT	12	15
pegHoxd13-2-PBS13	GAGGCATACATCTCCATGGA	GACTGGTAGACCTCCATGGAGATGTATG	13	15
pegHoxd13-2-PBS14	GAGGCATACATCTCCATGGA	GACTGGTAGACCTCCATGGAGATGTATGC	14	15
pegHoxd13-2-PBS15	GAGGCATACATCTCCATGGA	GACTGGTAGACCTCCATGGAGATGTATGCC	15	15
pegHoxd13-2-PBS16	GAGGCATACATCTCCATGGA	GACTGGTAGACCTCCATGGAGATGTATGCCT	16	15
pegHoxd13-2-PBS17	GAGGCATACATCTCCATGGA	GACTGGTAGACCTCCATGGAGATGTATGCCTC	17	15
pegAr-2-RT10	TCTCACTTGTGGCAGCTGCA	AAGACCTTGAAGCTGCCACAAGT	13	10
pegAr-2-RT11	TCTCACTTGTGGCAGCTGCA	GAAGACCTTGAAGCTGCCACAAGT	13	11
pegAr-2-RT12	TCTCACTTGTGGCAGCTGCA	AGAAGACCTTGAAGCTGCCACAAGT	13	12
pegAr-2-RT13	TCTCACTTGTGGCAGCTGCA	AAGAAGACCTTGAAGCTGCCACAAGT	13	13
pegAr-2-RT14	TCTCACTTGTGGCAGCTGCA	GAAGAAGACCTTGAAGCTGCCACAAGT	13	14
pegAr-2-RT15	TCTCACTTGTGGCAGCTGCA	TGAAGAAGACCTTGAAGCTGCCACAAGT	13	15
pegAr-2-RT16	TCTCACTTGTGGCAGCTGCA	TTGAAGAAGACCTTGAAGCTGCCACAAGT	13	16
pegAr-2-RT17	TCTCACTTGTGGCAGCTGCA	TTTGAAGAAGACCTTGAAGCTGCCACAAGT	13	17
pegAr-2-RT18	TCTCACTTGTGGCAGCTGCA	TTTTGAAGAAGACCTTGAAGCTGCCACAAGT	13	18
pegAr-2-RT19	TCTCACTTGTGGCAGCTGCA	CTTTTGAAGAAGACCTTGAAGCTGCCACAAGT	13	19
pegAr-2-RT20	TCTCACTTGTGGCAGCTGCA	TCTTTTGAAGAAGACCTTGAAGCTGCCACAAGT	13	20
pegAr-2-PBS10	TCTCACTTGTGGCAGCTGCA	GAAGAAGACCTTGAAGCTGCCACA	10	14
pegAr-2-PBS11	TCTCACTTGTGGCAGCTGCA	GAAGAAGACCTTGAAGCTGCCACAA	11	14
pegAr-2-PBS12	TCTCACTTGTGGCAGCTGCA	GAAGAAGACCTTGAAGCTGCCACAAG	12	14
pegAr-2-PBS13	TCTCACTTGTGGCAGCTGCA	GAAGAAGACCTTGAAGCTGCCACAAGT	13	14
pegAr-2-PBS14	TCTCACTTGTGGCAGCTGCA	GAAGAAGACCTTGAAGCTGCCACAAGTG	14	14
pegAr-2-PBS15	TCTCACTTGTGGCAGCTGCA	GAAGAAGACCTTGAAGCTGCCACAAGTGA	15	14
pegAr-2-PBS16	TCTCACTTGTGGCAGCTGCA	GAAGAAGACCTTGAAGCTGCCACAAGTGAG	16	14
pegAr-2-PBS17	TCTCACTTGTGGCAGCTGCA	GAAGAAGACCTTGAAGCTGCCACAAGTGAGA	17	14
**nicking sgRNA**	**spacer sequence**			
Hoxd13-1_+48	GATCCTTGGCACAGTACACC			
Hoxd13-2_+48	GATCCTTGGCACAGTACACC			
Hoxd13-3_+55	CCTTCGATTCTGAAACCAAA			
Hoxd13-4_+52	CCTTCGATTCTGAAACCAAA			
Hoxd13_+2	CCAGGACTGGTAGGCCTCCA			
Ar-1_+38	TCTCCCCGAAGCTGTTCCCC			
Ar-2_+49	AGGGGAAAATATCAGGAAGT			
Ar-3_+50	CTTCTTTTCCTTACCAGGCA			
Ar-4_+42	ACTACCCAAGTCCCCATAG			
Ar-5_+40	CTGTGCCCTTGGTCAAAAGG			

Supplementary Table S3 Sequences for plasmids construction.

Description	sequence
backbone_pegRNA-F	AGCTAGGTCTCCTTTTTTTAAAGAATTCTCGACCTCGAGAC
backbone_pegRNA-R	TCTCTCGGTCTCACGGTGTTTCGT
sg_scaffold-top	AGAGCTAGAAATAGCAAGTTAAAATAAGGCTAGTCCGTTATCAACTTGAAAAAGTGGCACCGAGTCG
sg_scaffold-bottom	GCACCGACTCGGTGCCACTTTTTCAAGTTGATAACGGACTAGCCTTATTTTAACTTGCTATTTCTAG

Supplementary Table S4 Primers used for mammalian cell genomic DNA amplification and targeted deep sequencing.

Description	Forward	Reverse
RNF2	ACGTCTCATATGCCCCTTGG	ACGTAGGAATTTTGGTGGGACA
Runx1	TCACAAACAAGACAGGGAACTG	AGATGTAGGGCTAGAGGGGTG
EMX1	CTATGTAGCCTCAGTCTTCC	CTCTCCGAGGAGAAGGCCAA
FANCF	ATGCTGCGCTTCAATGGCTA	TGAAACCTATTGTGCAACTC
KCNA1	ACATCTTCACAGACCCCTTC	CCACGATCTTGCCTCCAATT
SRD5A1	GGCATACCTGACAACAGTCCT	ATCCTCTCTTCCCAAGCACAG
SRD5A3	GCCTCCGTTTTGGGAACTTG	GGGAAGTCAGGAGCGCTAAC
VEGFA	CTTCTGGGCTGTTCTCGCTT	GTGAGCCTCTCTCCGGGTAC
Ar-1	TAATCTCCGAAGGCAGCAGCG	TCGTCCAGGGGAAGACCTTT
Ar-2	CGATGTCTGCCATCCACTGA	TCTGCTAGGCAAAAGAGAAGGG
Ar-3	GGAGAAAACTCCAATGCTGGC	TGCATCCCACATCCTCATTC
Ar-4	TTGAGATCCCGTCCTCTCTGT	CTCCATGTAGACTACCCAAGT
Ar-5	GGAGAAAACTCCAATGCTGGC	TGCATCCCACATCCTCATTC
Hoxd-1	TACACAAGTCCCTATCAGCAC	GCCTGGAATATAAGTAACCAC
Hoxd-2	TACACAAGTCCCTATCAGCAC	GCCTGGAATATAAGTAACCAC
Hoxd-3	CATCAAATTAGCCTTTGTCTC	TAAACTGTCTGTGGCCAACCT
Hoxd-4	CATCAAATTAGCCTTTGTCTC	TAAACTGTCTGTGGCCAACCT
deepseq-Ar-1	ATCACGCGATGTCTGCCATCCACTGA	TCTGCTAGGCAAAAGAGAAGGG
deepseq-Ar-2	CGATGTTCGATGTCTGCCATCCACTGA	TCTGCTAGGCAAAAGAGAAGGG
deepseq-Ar-3	TTAGGCAGCGATGTCTGCCATCCACTGA	TCTGCTAGGCAAAAGAGAAGGG
deepseq-Ar-4	TGACCAGCTCGATGTCTGCCATCCACTGA	TCTGCTAGGCAAAAGAGAAGGG
deepseq-Ar-5	ACAGTGCTCGATGTCTGCCATCCACTGA	TCTGCTAGGCAAAAGAGAAGGG
deepseq-Ar-6	GCCAATCGATGTCTGCCATCCACTGA	TCTGCTAGGCAAAAGAGAAGGG
deepseq-Ar-7	CAGATCTCGATGTCTGCCATCCACTGA	TCTGCTAGGCAAAAGAGAAGGG
deepseq-Ar-8	ACTTGAAACGATGTCTGCCATCCACTGA	TCTGCTAGGCAAAAGAGAAGGG
deepseq-Ar-9	GATCAGGGACGATGTCTGCCATCCACTGA	TCTGCTAGGCAAAAGAGAAGGG
deepseq-Ar-10	TAGCTTCCCGATGTCTGCCATCCACTGA	TCTGCTAGGCAAAAGAGAAGGG
deepseq-Ar-11	GGCTACCGATGTCTGCCATCCACTGA	TCTGCTAGGCAAAAGAGAAGGG
deepseq-Ar-12	CTTGTACGATGTCTGCCATCCACTGA	TCTGCTAGGCAAAAGAGAAGGG
deepseq-Ar-13	AGTCAACGATGTCTGCCATCCACTGA	TCTGCTAGGCAAAAGAGAAGGG
deepseq-Ar-14	AGTTCCACGATGTCTGCCATCCACTGA	TCTGCTAGGCAAAAGAGAAGGG
deepseq-Ar-15	ATGTCACGCGATGTCTGCCATCCACTGA	TCTGCTAGGCAAAAGAGAAGGG
deepseq-Ar-16	CCGTCCCGATGTCTGCCATCCACTGA	TCTGCTAGGCAAAAGAGAAGGG
deepseq-Ar-17	GTAGAGCCGATGTCTGCCATCCACTGA	TCTGCTAGGCAAAAGAGAAGGG
deepseq-Ar-18	GTCCGCACGATGTCTGCCATCCACTGA	TCTGCTAGGCAAAAGAGAAGGG
deepseq-hoxd-1	ATCACGTACACAAGTCCCTATCAGCAC	GCCTGGAATATAAGTAACCAC
deepseq-hoxd-2	CGATGTTTACACAAGTCCCTATCAGCAC	GCCTGGAATATAAGTAACCAC
deepseq-hoxd-3	TTAGGCAGTACACAAGTCCCTATCAGCAC	GCCTGGAATATAAGTAACCAC
deepseq-hoxd-4	TGACCAGCTTACACAAGTCCCTATCAGCAC	GCCTGGAATATAAGTAACCAC
deepseq-hoxd-5	ACAGTGCTTACACAAGTCCCTATCAGCAC	GCCTGGAATATAAGTAACCAC

deepseq-hoxd-6	GCCAATTACACAAGTCCCTATCAGCAC	GCCTGGAATATAAGTAACCAC
deepseq-hoxd-7	CAGATCTTACACAAGTCCCTATCAGCAC	GCCTGGAATATAAGTAACCAC
deepseq-hoxd-8	ACTTGAAATACACAAGTCCCTATCAGCAC	GCCTGGAATATAAGTAACCAC
deepseq-hoxd-9	GATCAGGGATACACAAGTCCCTATCAGCAC	GCCTGGAATATAAGTAACCAC
deepseq-hoxd-10	TAGCTTCCTACACAAGTCCCTATCAGCAC	GCCTGGAATATAAGTAACCAC
deepseq-hoxd-11	GGCTACTACACAAGTCCCTATCAGCAC	GCCTGGAATATAAGTAACCAC
deepseq-hoxd-12	CTTGTATACACAAGTCCCTATCAGCAC	GCCTGGAATATAAGTAACCAC
deepseq-hoxd-13	AGTCAATACACAAGTCCCTATCAGCAC	GCCTGGAATATAAGTAACCAC
deepseq-hoxd-14	AGTTCCATACACAAGTCCCTATCAGCAC	GCCTGGAATATAAGTAACCAC
deepseq-hoxd-15	ATGTCACGTACACAAGTCCCTATCAGCAC	GCCTGGAATATAAGTAACCAC
deepseq-hoxd-16	CCGTCCTACACAAGTCCCTATCAGCAC	GCCTGGAATATAAGTAACCAC
deepseq-hoxd-17	GTAGAGCTACACAAGTCCCTATCAGCAC	GCCTGGAATATAAGTAACCAC
deepseq-hoxd-18	GTCCGCATACACAAGTCCCTATCAGCAC	GCCTGGAATATAAGTAACCAC
peg-OT1	ATGGAAACGCTTGGGAAATG	TAACTGGCAGAAATTGGCTG
peg-OT2	TTAACCATACCCCTGCTCTG	CTTCAGCAGGACTAGCCAAG
peg-OT3	GAATGAGGCCAGTTGGAATT	ATCTGCCTGGAGACCAAAAC
peg-OT4	GGTCTTGATCTCCTAGCAAT	TAATCCTTTGCCTCAGACTC
peg-OT5	TTCAAGGCCAACCCACAGAG	TTCCTCTGATTAGGGAATGG
peg-OT6	CTGTGCTTGGTATATGTGGT	ATTTCTACATAGCCCTGGCT
peg-OT7	GCTCAATGTGTTTGCAGGAT	GGTTTCACTGCTATCTTGTG
peg-OT8	GAAGGCCGTGACTCCTGTAA	AACAACAGTCGGGAAGGATC
peg-OT9	TACGAGAGTGGCTTACAGGC	CACCTTCTGCTGGCAGTTTC
peg-OT10	TGAAGAGGCTCTTTGGAGTT	TCTCAGTCGCTGTAAGCTGT
nick-OT1	GGTATGAGTTGGGAAAGCAA	AAAGGGTGGTAGCAACCAAG
nick-OT2	TTGAATCTGACACCTTTCTG	ACTAGTGTTCCGGTGCGGCA
nick-OT3	GAGCACCTAGTAGAATGTGC	TCATACTGTCATAGTGCTGG
nick-OT4	GGATAGGATTTCAGGATGTT	GTGCATTAGGCACATGGACC
nick-OT5	GGCAAGATGGTCCTTCTGGT	CGTAACATGTCTACTTAGCA
nick-OT6	TGGCATGTTTTAGCTGCCAA	GGACAGTCCTATCTTGGAAG

Supplementary Table S5 Primes for *in vitro* transcription.

Description	sequence
IVT-Hoxd13-F	GATCCCTAATACGACTCACTATAGGGAGGCATACATCTCCATGGA
IVT-Hoxd13-1-R	AAAAAAAACATCTCCATGGAGGCCTACCAGTCGCACCG
IVT-Hoxd13-2-R	AAAAAAAACATCTCCATGGAGGTCTACCAGTCGCACCG
IVT-Ar-2-F	GATCCCTAATACGACTCACTATAGGAGTGTCTGTGTCCATGGGAT
IVT-Ar-2-R	AAAAAAAACTTGTGGCAGCTTCAAGGTCTTCTT
